# Radiological implications of crestal and subcrestal implant placement in posterior areas. A cone-beam computed tomography study

**DOI:** 10.4317/jced.56652

**Published:** 2020-09-01

**Authors:** Hilario Pellicer-Chover, Julio Rojo-Sanchís, Miguel Peñarrocha-Diago, José Viña-Almunia, David Peñarrocha-Oltra, Maria Peñarrocha-Diago

**Affiliations:** 1 DDS, PhD. Collaborating Professor of the Master of Oral Surgery and Implant Dentistry, Oral Surgery Unit, Department of Stomatology, Faculty of Medicine and Dentistry, University of Valencia, Spain; 2 DDS, Master in Oral Surgery and Implant Dentistry, Department of Stomatology, Faculty of Medicine and Dentistry, University of Valencia, Spain; 3 MD, PhD. Chairman of Oral Surgery and Director of the Master of Oral Surgery and Implant Dentistry, Oral Surgery Unit, Department of Stomatology, Faculty of Medicine and Dentistry, University of Valencia, Spain; 4 DDS, PhD. Associate Professor Oral Surgery, Oral Surgery Unit, Department of Stomatology, Faculty of Medicine and Dentistry, University of Valencia, Spain; 5 DDS, PhD. Assistant Professor, Oral Surgery Unit, Department of Stomatology, Faculty of Medicine and Dentistry, University of Valencia, Spain; 6 MD, PhD. Associate Professor Oral Surgery, Oral Surgery Unit, Department of Stomatology, Faculty of Medicine and Dentistry, University of Valencia, Spain

## Abstract

**Background:**

Subcrestal implant placement has been suggested as a method that could contribute to maintain the periimplant soft and hard tissues in comparison with crestal placement. The objective of this study was to investigate the relationship between implant placement at different depths in the alveolar bone and (a) the thickness of the buccal bone plate (BBP); and (b) crestal cortical bone thickness, based on the use of cone-beam computed tomography (CBCT).

**Material and Methods:**

A cross-sectional study was performed, analyzing CBCT scans from the database of the Oral Surgery Unit of the University of Valencia. Individuals with single missing teeth in posterior sectors were included. Two trained dentists used a software application to plan implant placement at four different depths from the bone crest (from 0-2 mm subcrestal). The thickness of the BBP was measured at each established depth, tracing a line from the implant platform to the outermost part of the facial alveolar bone, and the ratio between the implant platform and cortical bone thickness was calculated.

**Results:**

The study sample consisted of 64 patients. In the case of implants placed in a crestal position, the distance from the platform to the BBP was 1.99±1.10 mm. This distance increased significantly (*p*<0.001) with the planned implant placement depth, reaching an average of 2.90±1.22 mm when placement was 2 mm subcrestal. Subcrestal implant placement at this depth implied surpassing the cortical bone in 91% of the cases.

**Conclusions:**

Radiological planning of implant placement in a subcrestal position results in a greater distance from the implant platform to the BBP. In general terms, planning implant placement at a depth of 2 mm subcrestal surpassed the cortical bone in 91% of the cases.

** Key words:**Subcrestal implant, cortical bone thickness, buccal bone plate, cone-beam computed tomography.

## Introduction

The amount and quality of bone are crucial factors for long-term success in dental implant treatments. In this regard, adequate bone volumes are not always available, and in such cases guided bone regeneration techniques or crestal bone osteoplasty may prove necessary prior to implant placement (1).

The stability of implant health over the long term is dependent upon achieving optimal three-dimensional implant positioning within the available bone dimensions, and the maintenance of adequate buccal bone over the buccal implant surface. Bone remodeling or resorption can be a physiological or pathological process occurring in response to trauma, or to physical, chemical or microbiological events in the vicinity of the implant site. Buccal bone is particularly sensitive to such bone changes (2). Alveolar buccal bone anatomy has been well studied, and in a very large percentage of cases the buccal bone layer measures less than 1 mm in thickness (3-5). In these thin bone phenotypes, the first buccal coronal millimeters are only composed of bundle bone - a tooth dependent structure that is reabsorbed following tooth extraction (6,7). However, in the case of dental implants there is no agreement as to what minimum amount of bone is needed to secure stability or regeneration. Spray *et al.* (8). Black, reported that as the bone thickness approaches 1.8 to 2 mm, crestal bone loss decreases significantly and some bone gain is seen. There is not much more evidence in support of this observation, however.

Subcrestal implant placement has been proposed as a method that could reduce bone loss, since the likelihood of finding an implant in a subcrestal position in the course of follow-up is greater when the implant is placed subcrestal from the start, i.e., on the day of surgery (9-11). A number of hypotheses have been proposed to explain this. A classical hypothesis is the restoration of biological thickness (12), which in the case of a thin peri-implant mucosa would take place at the expense of bone resorption. Other possible explanations are referred to peri-implant bone volume and quality, as when the implant is placed in a crestal position, the implant platform (IP) is located entirely in cortical bone. While osseointegration may be fast in the area of bone marrow and loosely trabecular bone, the osseointegration may require longer periods of time in areas of compact bone owing to the fact that bone resorption may precede new bone formation (13). On the other hand, old bone provided mechanical stability of the implant during the first weeks of healing.

*In vitro* anatomical studies (14) have found that after physiological remodeling of the maxillae, alveolar bone often acquires a truncoconical anatomy, being narrower at its most coronal portion and gradually increasing in thickness towards the most apical part. Taking advantage of this anatomical particularity, implants placed subcrestal on the day of surgery could result in increased peri-implant bone thickness.

Ko *et al.* (15) found crestal cortical bone thickness at dental implant sites to vary in different regions of the jawbone – the mean thickness in the mandible and posterior maxilla being 1.07±0.47 mm and 0.75±0.35 mm, respectively. The placement of implants in a subcrestal position could imply surpassing the cortical bone as the insertion depth increases. Many finite element analyses (16) have found that placing an implant surrounded by trabecular bone results in better load distribution and lesser peri-implant stress. In this regard, *in vitro* studies (17-19) observed that implants placed in cortical or cancellous bone presented different healing patterns, due to differences in the density of the bone in primary contact with the implant surfaces. Cortical bone leads to a delay in bone formation, while cancellous bone can allow a rapid bone apposition thanks to the presence of medullary spaces interposed between the trabeculae.

To the best of our knowledge, no studies have explored the relationships among apico-coronal positioning of the implant, the thickness of the buccal bone plate, and crestal cortical bone thickness. The present cross-sectional study was designed to investigate the relationship between implant placement at different depths in the bone alveolar and (a) the thickness of the buccal bone plate; and (b) crestal cortical bone thickness, based on the use of cone-beam computed tomography (CBCT).

## Material and Methods

-Study design

A cross-sectional study was carried out, analyzing CBCT scans from the database of the Oral Surgery Unit of the University of Valencia (Valencia, Spain), corresponding to patients subjected to dental implant treatment between May 2013 and November 2017. The study protocol complied with the ethical principles of the World Medical Association Declaration of Helsinki, and was approved by the local Research Ethics Committee (Ref. H1365580155510). This article was written following the STROBE statement for improving the quality of observational studies.

-Sample selection

The following inclusion criteria were established: patients with single missing teeth in posterior sectors (premolars and molars), with a fully healed alveolar process (20), intact cortical bone layers, and a thickness in the coronal portion of 6 mm or more for the placement of an implant measuring 4.0 mm in diameter and 10 mm in length (21). The CBCT studies were required to present radiological splints with a radiopaque marker indicating the correct position of the tooth needing rehabilitation. Patients with Cawood & Howell (22) type IV or V atrophy and who required bone augmentation procedures for implant placement were excluded, as were those cases involving poor quality CBCT images (presence of artifacts and interferences due to previous treatments in the form of adjacent implants, crowns or bridges) that would complicate data interpretation.

Radiographic analysis

The CBCT scans were obtained using the NewTom 3G system (Verona, Italy), and the images were acquired by means of NNT software (version 2.17), with a voxel size of 150 mSv, 90 kV, 10.0 mA and a field of view (FOV) of 4 x 4 cm. All the images were analyzed with the same computer and screen (Eizo Nanao Flexscan, resolution 1280 x 1024 pixels).

**Figure 1:**
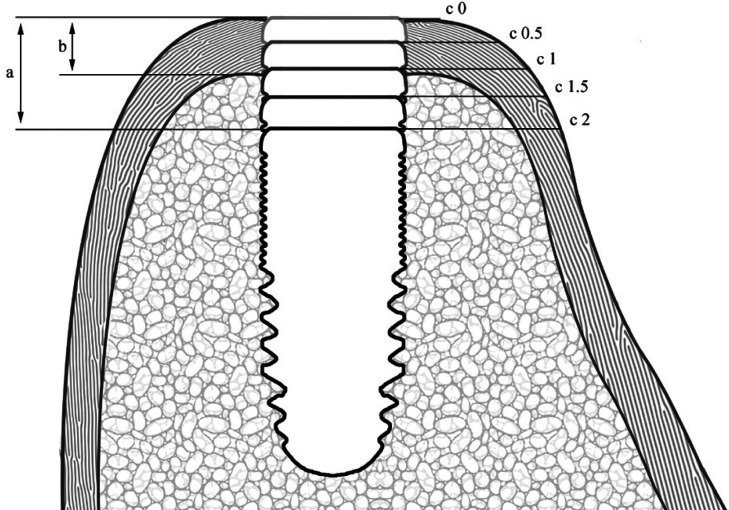
Graphic representation of the study variables: a) planned implant depth; b) thickness of the crestal cortical bone, c) distance from the implant platform to the outermost part of the buccal bone plate (IP-BBP) at the different planned implant depths.

Two trained and calibrated investigators (J.R.S. and H.P.C.) independently conducted radiological planning and measurement of the variables. The edentulous gap was first located in the axial plane, and then a section was obtained in the sagittal plane taking as reference the radiopaque marker of the radiological splint. Planning of the implant was made in a coronal section, locating the implant in different apico-coronal positions with respect to the alveolar bone crest: crestal position, 0.5 mm subcrestal, 1 mm subcrestal, 1.5 mm subcrestal and 2 mm subcrestal. In each of these positions, measurement was made of the distance from the implant platform (IP) to the outermost portion of the buccal bone plate (BBP). The thickness of the crestal cortical bone was recorded in the central zone of the edentulous gap (15). The mean values of both examiners were used for the analysis (Fig. 1).

-Statistical analysis

The intraclass correlation coefficient (ICC) (0.985) indicated very high inter-examiner reproducibility. Normal distribution of the different study variables was checked using the Kolmogorov-Smirnov test. An analysis of variance (ANOVA) general linear model of repeated measures was used to determine whether the mean thickness of the bone layer was similar at different insertion depths or not. As post hoc test, Bonferroni correction was applied to avoid propagation of type I error. A one-way ANOVA general linear model with inter-subjects factor was used for the position, and a two-way ANOVA was used to analyze the combined effect and interaction of the position and arch with bone thickness. Estimation of survival curves for the event “surpassing the cortical bone” was applied, with the log-rank test for comparison according to the position and/or arch. A Cox regression model was used to estimate the hazard ratio (HR). The level of statistical significance considered was 5% (α=0.05). The proposed statistical methodology, with a level of confidence of 95% and considering an effect size to be detected f=0.15 (medium-small), afforded a statistical power of 87% in contrasting intra-subject effects (differences between depth levels).

## Results

The study sample consisted of 64 edentulous gaps corresponding to 18 maxillary premolars, two mandibular premolars, 17 maxillary molars and 27 mandibular molars. Dental CBCT images were collected from 64 patients (35 women and 29 men) with a mean age of 57.0±10.4 years. The mean thickness of the alveolar process was 7.44±1.49 mm at the most coronal portion (7.30±1.44 mm in maxilla and 7.62±1.56 mm in mandible).

**Figure 2:**
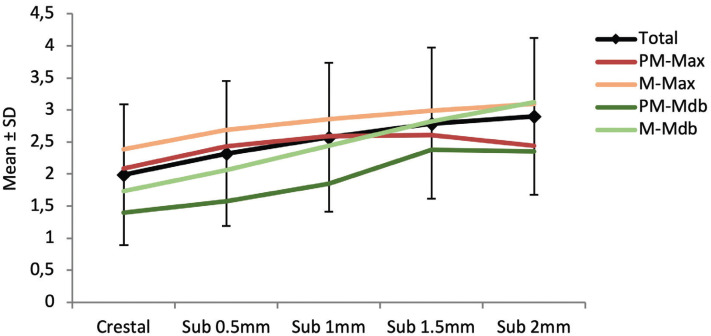
Mean IP-BBP distance (in mm) according to implant depth. PM: premolar; M: molar; Max: maxilla; Mdb: mandible.

The implants planned in a crestal position showed a mean IP-BBP distance of 1.99±1.10 mm, while at the maximum planned insertion depth (2 mm) the distance was 2.90±1.22 mm. The distance increased significantly with the implant placement depth (*p*<0.001), though the Bonferroni multiple comparison test showed no differences between planned depths of 1.5 to 2 mm (*p*=0.471). Table 1 and Figure 2 show the mean IP-BBP distances according to depth, arch (maxilla or mandible) and position (premolar or molar). In the mandible, the IP-BBP distance increased progressively with implant depth (*p*=0.001). In contrast, in the maxilla we observed an attenuation of the IP-BBP distance from a depth of 1 mm. With regard to implant position, the increase in distance was more notorious in the case of the molars (*p*=0.002), since stability was observed in the premolars from 1 mm.

**Table 1:**
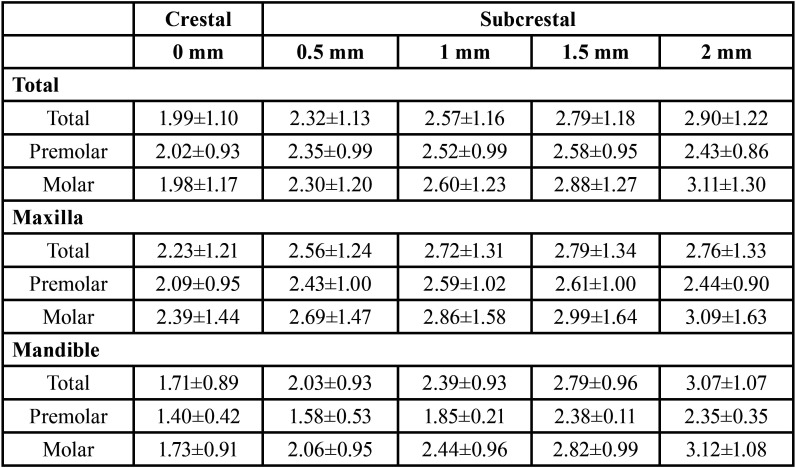
Implant platform-BBP distance (in mm) according to the apico-coronal position of the implant (mean ± standard deviation), arch and position.

**Figure 3:**
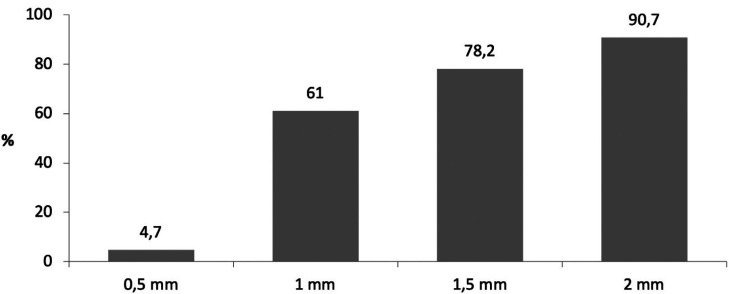
Percentage of implants that surpass the cortical bone layer according to the planned insertion depth.

The mean thickness of the crestal cortical bone was 1.16±0.97 mm (Table 2). Figure 3 shows the percentage of planned cases that surpassed the cortical layer according to the depth of insertion. The mean thickness was 0.93±0.75 mm in the maxilla and 1.44±1.15 mm in the mandible – the difference being statistically significant (*p*=0.008). The mean thickness in premolars and molars was 0.98±0.56 mm and 1.25±1.11 mm, respectively – the difference being nonsignificant (*p*=0.376).

**Table 2:**
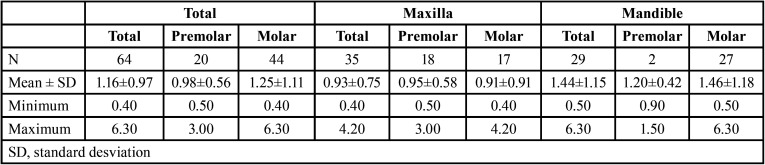
Crestal cortical bone thickness (in mm) at the dental implant sites according to the arch and position involved.

## Discussion

The aim of the present study was to determine how implant placement at different depths in the alveolar bone influences a series of characteristics of peri-implant bone that could be related to the resorption pattern, namely the thickness of the buccal bone plate and crestal cortical bone thickness. A number of recent studies (9-11) have associated subcrestal positioning of dental implants to lesser periimplant marginal bone. A number of hypotheses have been proposed to explain this observation. One of the most widely accepted hypotheses is related to the thickness of the peri-implant mucosa and the restoration of biological thickness (23). Another possible explanation refers to the characteristics of peri-implant bone. In the present study, implants placed in a crestal position, the distance from the implant platform to the buccal bone plate was 1.99±1.10 mm. This distance increased significantly with the planned implant placement depth, reaching an average of 2.90±1.22 mm when placement was 2 mm subcrestal.

Based on dental cone-beam computed tomography (CBCT) images, we selected radiological studies of patients requiring single implant placement and which presented radiological splints – thereby facilitating correct guided implant placement and resulting in high reproducibility between examiners (ICC=0.985). We selected edentulous alveolar crests with a minimum thickness of 6 mm, so that both the crestal and subcrestal implants had sufficient bone volume for placing a standard implant measuring 4 mm in diameter and 10 mm in length, without the need for bone augmentation measures (21) Radiological studies involving edentulous zones in anterior sectors were excluded. In most cases, implant placement in anterior sectors requires the use of bone augmentation techniques, and this could influence initial planning of the implant.

*Pi*etrokovski *et al.* (14) examined the bone tissue characteristics of edentulous arches and residual ridges in different regions of 123 human edentulous dry bone specimens. With the premise that the implant protocols require the cervical implant neck to be completely embedded in the bony residual crest, the results showed a high percentage of narrow alveolar processes – a fact that could compromise the success of implant treatment. Aloy-Prosper *et al.* (24) found the most frequent peri-implant defects during implant surgery to involve dehiscence of the buccal layer – a situation requiring the use of a guided bone regeneration technique.

Morphological bone changes after tooth extraction have been well studied by Araujo & Lindhe6 in Beagle dogs. The mesial, distal and lingual/palatal aspects hardly undergo remodeling, though a vertical loss of 2.2 mm has been reported at the buccal aspect (25). In most cases such remodeling confers a truncoconical shape in which the most coronal portion narrows in comparison with the most basal part. This anatomical feature results in greater thickness of the alveolar process on advancing in depth in the apical direction. In consequence, there may be a greater presence of peri-implant bone and a longer distance from the body of the implant to the external cortical layers when implantation is made below the bone crest. In our study, the implants planned in a subcrestal position presented a greater IP-BBP distance than the implants placed in a crestal position – the difference being statistically significant. The minimum BBP thickness required to avoid vertical crest resorption has not been established to date (26). A publication by a panel of experts and master clinicians in implantology showed that once the implant osteotomy was performed, an ideal BBP thickness of 2 mm proved advisable in order to secure an optimum biological and esthetic outcome (27). This fact could avoid crestal bone loss and future implant dehiscence, which are the most frequent bone defects present when peri-implantitis becomes established (28).

Another procedure that could contribute to increase the thickness of the alveolar process is crestal bone osteoplasty. Hudieband & Kasugai (21) conducted a finite element analysis examining the biomechanical effects of crestal bone osteoplasty in narrow edentulous crests before dental implant placement. Although osteoplasty of the bone crest reduced tension at the implant neck, the elimination of the cortical bone and exposure of the bone trabecular resulted in increased tension of the peri-implant bone, which in turn could contribute to bone loss.

The mean crestal cortical bone thickness values at the dental implant sites were 0.93±0.75 mm in maxilla and 1.44±1.15 mm in mandible. These results suggest the need for a greater planned implant depth in mandibular zones in order to surpass the cortical bone and reach the trabecular bone. In contrast, the differences between premolar (0.98±0.56 mm) and molar positioning (1.25±1.11 mm) were similar and showed no statistically significant differences. The planning of implants placed 2 mm subcrestal implied surpassing the cortical bone in 90% of the cases – the implant platform being positioned in trabecular bone. Experimental studies in animals (17,18) have reported a greater percentage bone-to-implant contact (BIC) in trabecular bone compared with cortical bone. Wang *et al.* (29) likewise in an experimental study in animals, observed a greater presence of osteoprogenitor cells in type II and III bone, resulting in faster production of new bone than in type I bone. In this respect, Sotto-Maior *et al.* (30) in a finite element analysis, recorded a decrease in compressive peri-implant tension when the implants were placed subcrestal (34.1 MPa), becoming completely surrounded by trabecular bone. In contrast, implants placed crestal showed greater peri-implant tension (199.2 MPa). Although definitive clinical evidence is lacking, peri-implant tensions of between 100-130 MPa could cause bone resorption secondary to overload (30).

The present study has limitations. A first consideration is its design, with implant placement being planned using three-dimensional planning software. This complicates extrapolation of the results to the clinical setting, and only allows us to speculate about how the implants would behave *in vivo*. Nevertheless, the design employed allowed us to establish intra-subject comparisons, since the scenarios could be planned in the same coronal section of the edentulous gap. Due to the great variability of the anatomy of the maxillae, this would have been very difficult to do between subjects. A second limitation is the difficulty of applying the methodology, since the measurements were made with 0.5-mm increments. These increments were easy to measure with the planning application, but it could prove complicated to extrapolate such small measurements in a clinical procedure. On the other hand, the sample size corresponding to implants placed in premolar gaps within the mandible was too small to allow the firm definition of possible interactions.

Despite the limitations of the present study, the results obtained suggest that planning dental implants in a subcrestal position would result in greater peri-implant buccal bone thickness. Moreover, the planning of implant placement at 1 mm, 1.5 mm and 2 mm subcrestal was seen to surpass the cortical bone in 61%, 78% and 91% of the cases, respectively.
